# Micro-Motion Extraction for Marine Targets by Multi-Pulse Delay Conjugate Multiplication and Layered Tracking

**DOI:** 10.3390/s23083837

**Published:** 2023-04-09

**Authors:** Tong Mao, Yi Zhang, Kaiqiang Zhu, Houjun Sun

**Affiliations:** Beijing Key Laboratory of Millimeter Wave and Terahertz Techniques, School of Integrated Circuits and Electronics, Beijing Institute of Technology, Beijing 100081, China; maotong@bit.edu.cn (T.M.); 3120160357@bit.edu.cn (K.Z.); sunhoujun@bit.edu.cn (H.S.)

**Keywords:** micro-doppler, micro-motion, multi-pulse delay conjugate multiplication, extended target tracking, layered tracking

## Abstract

The detection and recognition of marine targets can be improved by utilizing the micro-motion induced by ocean waves. However, distinguishing and tracking overlapping targets is challenging when multiple extended targets overlap in the range dimension of the radar echo. In this paper, we propose a multi-pulse delay conjugate multiplication and layered tracking (MDCM-LT) algorithm for micro-motion trajectory tracking. The MDCM method is first applied to obtain the conjugate phase from the radar echo, which enables high-precision micro-motion extraction and overlapping state identification of extended targets. Then, the LT algorithm is proposed to track the sparse scattering points belonging to different extended targets. In our simulation, the root mean square errors of the distance and velocity trajectories were better than 0.277 m and 0.016 m/s, respectively. Our results demonstrate that the proposed method has the potential to improve the precision and reliability of marine target detection through radar.

## 1. Introduction

Micro-motion plays an important role in radar target detection and classification [1]. The vibration of a marine target caused by wave slapping is a unique feature that can be exploited for target identification and voyage monitoring [2]. Extracting vibration parameters can be challenging when performing long-distance radar detection, since the projection of micro-motion on the radar line-of-sight may be weak. The phase-derived measurement technique has been employed to achieve high-precision range estimation and motion feature extraction [3,4,5,6], which utilizes the principle that a half-wavelength translation of a target induces a 2π phase delay in radar echo.

In the range domain of wideband radar echo, the marine vessel can be regarded as an extended target composed of strong scattering points. Extended target detection is studied in [7,8,9]. In order to capture the target motion variation over time, the target tracking method was introduced. The Bayesian filtering framework is commonly used, including the Kalman filter (KF) [10] and the particle filter [11]. Moreover, the tracking problem can be divided into sub-problems of association between adjacent points and be solved through the dynamic programming method [12,13]. In long-distance scenes, the tangential coverage range of the radar beam may reach several hundreds of meters, and there may exist multiple targets in the radar sight. The main challenge in multiple extended target tracking is correctly associating different targets. For the multi-target tracking issue, the pairing between tracks and measurements is usually achieved through data association algorithms, such as nearest neighbor [14] and joint probabilistic data association [15]. In [16], the multiple points were separated into several groups, and the centers of the groups were tracked through the probability hypothesis density filter. In [17], the multi-Bernoulli filter algorithm was adopted to track the centers of groups.

However, when tracking the overlapping extended targets, current methods rely on motion model predictions or prior knowledge of target movements. It is difficult to directly acquire the overlapping state between targets. Additionally, existing extended target tracking methods primarily focus on tracking isolated points or the center of entire targets, without fully utilizing the motion correlation between scattering points, which reflects the structure of the extended target and offers new insights for target characteristic extraction. There is a pressing need to explore how to use the micro-motion of scattering points to enhance extended target tracking.

To address the problems, we proposed an extended target tracking algorithm based on the multi-pulse delay conjugate multiplication (MDCM) and layered tracking (LT). First, we analyzed the micro-motion features of marine targets and established the radar echo model. Then, the MDCM method was applied to calculate the conjugate phase, which was further used to differentiate the coincident points (superimposed by multiple scattering points) and independent points. With the assistance of independent points, the conjugate phases of the coincident points were corrected, and LT was finally achieved.

The reminder of this paper is organized as follows. Section 2 describes the movement model and the radar echo simulation of the marine target. Section 3 presents the MDCM-LT method. In Section 4, the proposed algorithm is verified through the simulation and the experiment. Furthermore, Section 5 concludes this paper.

## 2. Marine Target Micro-Doppler Modeling

### 2.1. Micro-Motion Model

According to sea-keeping theory, the stability and seaworthiness of a marine target are related to its size, shape, and weight distribution. Furthermore, various targets may exhibit distinct motion characteristics under identical sea conditions.

Linear strip theory [18] assumes the amplitude of the marine target motion is proportional to the amplitude of the ocean wave. The ocean wave forms from a superposition of regular waves as

(1) $$\xi \left(t\right)=\sum_{i=1}^{\infty }\xi_{i}\cos\left(\omega_{i}t+\varphi_{i}\right)$$

where $\xi_{i}$, $\omega_{i}$, and $\phi_{i}$ denote the amplitude, the angular frequency, and the phase of the *i*-th wave, respectively.

The micro-motion of a marine target encompasses three translational and three rotational degrees of freedom, and it can be expressed as

(2) $$a_{u}\left(t\right)=\sum_{i=1}^{\infty }{\mathrm{Rao}}_{u}\left(\omega_{i}\right)\xi_{i}\cos\left(\omega_{i}t+\varphi_{i}\right)$$

where *u* ranges from one to six, thereby representing the target movements in six degrees of freedom. Furthermore, the response amplitude operator, ${\mathrm{Rao}}_{u}\left(\omega_{i}\right)$, denotes the ratio between the movement amplitude to the regular wave amplitude at frequency $\omega_{i}$. In sea states of medium to low intensity, the movements along the longitudinal direction (heaving and pitching) are particularly influential on the micro-motion characteristics of the marine target. To simplify the vessel motion modeling, we focused on heaving and pitching movements.

**Figure 1:**
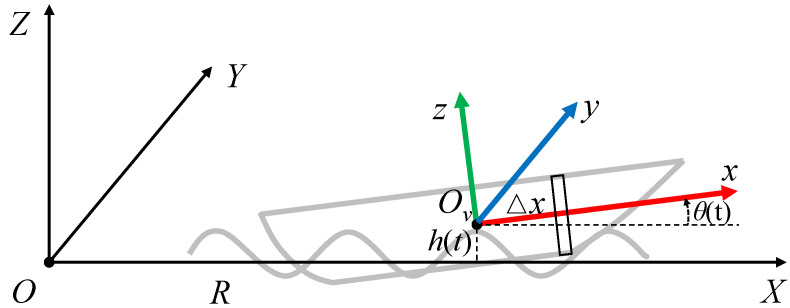
The coordinate systems of the vessel.

We adopted the microtome section theory [19] to create the micro-motion model of the marine vessel. Two coordinate systems are established: the global coordinate system *O*-XYZ and the vessel-fixed coordinate system $O_{v}$-xyz (shown in Figure 1). The radar is positioned at point *O*, while the longitudinal axis of the vessel is oriented along the +X axis. We assume that the coordinate of the vessel center, $O_{v}$, is (R,0,0). h(t) and θ(t) denote the height variation caused by heaving and the pitch angle of pitching, which are expressed as

(3) $$\begin{array}{c} h\left(t\right)=\sum_{i=1}^{\infty }{\mathrm{Rao}}_{h}\left(\omega_{i}\right)\xi_{i}\cos\left(\omega_{i}t+\varphi_{i}\right)\\ \theta \left(t\right)=\sum_{i=1}^{\infty }{\mathrm{Rao}}_{\theta }\left(\omega_{i}\right)\xi_{i}\cos\left(\omega_{i}t+\varphi_{i}\right) \end{array}$$

where ${\mathrm{Rao}}_{h}\left(\omega_{i}\right)$ and ${\mathrm{Rao}}_{\theta }\left(\omega_{i}\right)$ are the response amplitude operators of heaving and pitching, respectively.

The vessel is separated into multiple sections along its longitudinal direction, and the coordinate of each section is calculated as

(4) $$\begin{array}{c} x^{\prime }=R+\Delta x\cos\theta \left(t\right)\\ y^{\prime }=0\\ z^{\prime }=h\left(t\right)+\Delta x\sin\theta \left(t\right) \end{array}$$

where Δx is the distance between the section and $O_{v}$.

Then, the distance between the section and the radar is calculated by using the following equation:

(5) $$\begin{array}{c} R^{\prime }\left(t\right)={\left\{{\left[R+\Delta x\cos\theta \left(t\right)\right]}^{2}+{\left[h(t)+\Delta x\sin\theta (t)\right]}^{2}\right\}}^{\frac{1}{2}} \end{array}$$

By calculating the derivative of Equation (5) with respect to time, the velocity of the section is

(6) $$v\left(t\right)=\frac{1}{b}\left\{\left[h(t)-\Delta x\sin\theta (t)\right]\frac{dh(t)}{dt}+\Delta x\left[h(t)\cos\theta (t)-R\sin\theta (t)\right]\frac{d\theta (t)}{dt}\right\}$$

where $b=\sqrt{{\left(R+\Delta x\cos\theta \right)}^{2}+{\left(h\left(t\right)+\Delta x\sin\theta \right)}^{2}}$.

Due to Δx and h(t) being much smaller than *R*, *b* could be approximated as *R*. Based on Equation (6), the velocity difference between two sections with interval $\Delta x_{1}$ and $\Delta x_{2}$ is calculated as follows

(7) $$\begin{array}{c} \begin{array}{cc} \Delta v & =v_{\Delta x_{1}}\left(t\right)-v_{\Delta x_{2}}\left(t\right)\\ \; & \approx \frac{1}{R}\left[h\left(t\right)\cos\theta \left(t\right)\frac{d\theta (t)}{dt}-R\sin\theta \left(t\right)\frac{d\theta (t)}{dt}-\sin\theta \left(t\right)\frac{dh(t)}{dt}\right]\left(\Delta x_{1}-\Delta x_{2}\right) \end{array} \end{array}$$

In Equation (7), θ(t) and h(t) are variables associated with the overall vessel movement, and do not change with Δx. Therefore, the difference in velocities between sections depends linearly on their relative position.

### 2.2. Radar Echo Model

The radar waveform is the chirp pulse signal with carrier frequency $f_{c}$. We regard the vessel sections as scattering points and construct the radar echo by superimposing the echoes from strong scattering points as follows

(8) $$s_{r}\left(t\right)=\sum_{i=1}^{I}\sigma_{i}\mathrm{rect}\left[\frac{t-2R_{i}^{\prime }\left(t\right)/c}{T_{w}}\right]\exp\left\{j2\pi \left[f_{c}\left(t-\frac{2R_{i}^{\prime }\left(t\right)}{c}\right)+\frac{1}{2}K{\left(t-\frac{2R_{i}^{\prime }\left(t\right)}{c}\right)}^{2}\right]\right\}$$

where *I* denotes the total number of the scattering points, rect(·) is the rectangular function, $T_{w}$ is the pulse width, *K* is the modulation ratio, *c* is the microwave speed, and $\sigma_{i}$ and $R_{i}^{\prime }\left(t\right)$ represent the scattering amplitude and the distance of the *i*-th scattering point, respectively.

The matched filtering is applied to the radar echo to obtain the range profile of the vessel. The reference signal in the frequency domain is denoted as H(k), which is calculated by applying Fourier transform on the transmitting signal. The output of the matched filter is calculated as follows

(9) $$\begin{array}{c} \begin{array}{cc} x\left(t\right) & =\mathrm{IFFT}\left[S_{r}\left(k\right)\cdot H^{*}\left(k\right)\right]\\ \; & =\sum_{i=1}^{I}A_{i}\mathrm{sinc}\left[B\left(t-\frac{2R_{i}^{\prime }\left(t\right)}{c}\right)\right]\exp\left[-j\frac{4\pi R_{i}^{\prime }\left(t\right)}{\lambda }\right] \end{array} \end{array}$$

where IFFT(·) denotes the inverse fast Fourier Transform process, $S_{r}\left(k\right)$ is the Fourier transforms of $s_{r}\left(t\right)$, $H^{*}\left(k\right)$ is the conjugate of H(k), $A_{i}$ is the amplitude of the output result, *B* is the bandwidth of the chirp signal, and λ is the wavelength.

The Doppler frequency of the *i*-th scattering point could be aquired by calculating the phase derivation of the range profile in Equation (9) as

(10) $$f_{d,i}\left(t\right)=-\frac{2}{\lambda }\frac{dR_{i}^{\prime }\left(t\right)}{dt}$$

## 3. Micro-Motion Tracking for Extended Target

### 3.1. Micro-Motion Feature Extraction through MDCM

To simplify the analysis, we focused on the micro-motion of a single scattering point. The amplitude and envelope terms in Equation (9) are denoted as $A_{r}$, and the range profile of the *m*-th pulse can be expressed as

(11) $$x_{i}\left(m,t_{n}\right)=A_{r}\exp\left[-j\frac{4\pi R_{i}^{\prime }\left(t_{m}\right)}{\lambda }\right]+n\left(t_{m}\right)=A_{r}\exp\left[-j\frac{4\pi R_{i}^{\prime }\left(mT_{r}+t_{n}\right)}{\lambda }\right]+n\left(mT_{r}+t_{n}\right)$$

where $t_{n}$ is the fast time in pulse, $t_{m}$ represents the sampling time in the *m*-th pulse, $T_{r}$ is the pulse repetition time, $R^{\prime }\left(t_{m}\right)$ is the distance between the point and the radar at time $t_{m}$, and $n(t_{m})$ denotes the echo of the environment clutter.

The approximation of $R^{\prime }\left(t_{m}\right)$ in Equation (5) can be calculated using the first-order Taylor expansion as

(12) $$\begin{array}{cc} R^{\prime }\left(t_{m}\right) & ={\left\{R^{2}+h{\left(t_{m}\right)}^{2}+\Delta x^{2}+2R\cos\theta \left(t_{m}\right)\Delta x+2h\left(t_{m}\right)\sin\theta \left(t_{m}\right)\Delta x\right\}}^{\frac{1}{2}}\\ \; & \approx R+\frac{1}{2R}\left[2R\cos\theta \left(t_{m}\right)\Delta x+h{\left(t_{m}\right)}^{2}+\Delta x^{2}+2h\left(t_{m}\right)\sin\theta \left(t_{m}\right)\Delta x\right] \end{array}$$

Since Δx and $h(t_{m})$ are much smaller than *R*, the last three terms in the square brackets in Equation (12) can be neglected for analysis. Then, $R^{\prime }\left(t_{m}\right)$ can be approximated as

(13) $$R^{\prime }\left(t_{m}\right)\approx R+\cos\theta \left(t_{m}\right)\Delta x$$

where $\theta (t_{m})$ represents the pitch angle at time $t_{m}$.

$\theta (t_{m})$ varies slowly over pulses. Therefore, we assume $\theta (t_{m})$ to be linear during processing time with a velocity $v_{\theta }$. Substitute Equation (13) into Equation (11), and calculate the *l*-pulse MDCM as

(14) $$\begin{array}{cc} y(m+l,T_{n}) & =x_{r}\left(m+l,t_{n}\right)\cdot x_{r}^{*}\left(m,t_{n}\right)\\ \; & =A_{c}\exp\left\{j\frac{8\pi \Delta x}{\lambda }\sin\frac{lv_{\theta }T_{r}}{2}\sin\left[\theta \left(t_{m}\right)+\frac{v_{\theta }T_{r}}{2}\right]\right\} \end{array}$$

where $A_{c}$ is the amplitude, and *l* is the number of delayed intervals.

In Equation (14), the phase term is named as the conjugate phase. The range of the phase after the MDCM process is constrained by $\sin\left(lv_{\theta }T_{r}/2\right)$, which is named as the reduction ratio η. By changing the delayed interval *l*, we can manipulate the value of η and decrease the phase variation caused by micro-motion, thereby unwrapping the ambiguity phase. $lv_{\theta }T_{r}$ approaches zero when *l* does not exceed a few hundred, and $T_{r}$ is at the microsecond level. In such circumstances, η is approximately equivalent to $lv_{\theta }T_{r}/2$, and is linearly proportional to *l*.

### 3.2. Layered Tracking of Extended Target

**Figure 2:**
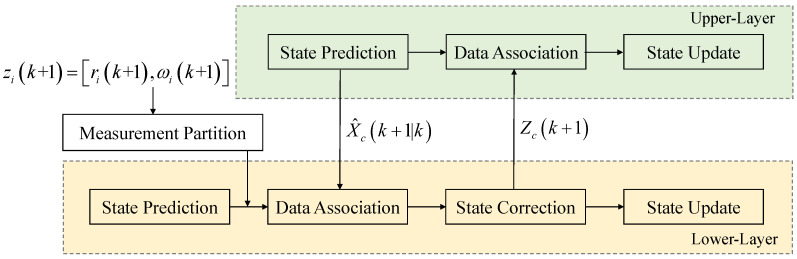
The framework of the layered tracking.

In this subsection, the tracking of the extended target was divided into two layers: the center tracking in the upper layer, and the scattering points tracking in the lower layer. In each time step, the measurement points $\left\{z_{i}\left(k+1\right)\right\}$ were initially partitioned into sets belonging to different extended targets. The wrong conjugate phases in $\left\{z_{i}\left(k+1\right)\right\}$ will be corrected on the basis of upper layer state prediction, ${\hat{X}}_{c}\left(k+1|k\right)$. Then, the average of $z_{i}\left(k+1\right)$ will be adopted as the measurement values of upper layer, $Z_{c}\left(k+1\right)$. The tracking process of the lower layer and the upper layer were both implemented using the KF method. The framework of the proposed LT method is depicted in Figure 2. The details of the LT algorithm are explained in the following parts.

#### 3.2.1. Measurement Partition

The appropriate partitioning of measurements is the prerequisite for effective tracking. We employed the distance partitioning principle [20] to divide the measurements at the initial frame. The Mahalanobis distance between two measurements, $z_{i}$ and $z_{j}$, is calculated as follows

(15) $$d_{i,j}={\left[{\left(z_{i}-z_{j}\right)}^{T}R\left(z_{i}-z_{j}\right)\right]}^{\frac{1}{2}}$$

where R is the distance weight matrix. When $d_{i,j}$ is smaller than the empirical threshold, the two measurements will be considered to belong to the same extended target.

When the scattering points of two extended targets coincide in the range dimension, the measurement partition becomes difficult, as more than one scattering points merge into one measurement, which we name the coincident point. To simplify the analysis, we assumed that a coincident point is superimposed by two scattering points with constant amplitudes. The phase of the coincident point, $\phi_{\Sigma }$, could be calculated as

(16) $$\phi_{\Sigma }=d\phi_{A}+e\phi_{B}$$

where $\phi_{A}$ and $\phi_{B}$ are the phases of the two points, respectively. *d* and *e* represent the proportion of $\phi_{A}$ and $\phi_{B}$ in $\phi_{\Sigma }$, respectively.

The MDCM under two delayed intervals, $l_{1}$ and $l_{2}$, is applied to the coincident point, and the conjugate phases are denoted as $\phi_{\Sigma ,1}$ and $\phi_{\Sigma ,2}$, respectively. Here, $l_{1}$ is small enough that the conjugate phases of two points are unambiguous, while $l_{2}$ is larger, and the phases are aliased. Let $\phi_{A,2}$ and $\phi_{B,2}$ denote the conjugate phases of the two scattering points under interval $l_{2}$; $\phi_{\Sigma ,2}$ can be calculated as

(17) $$\phi_{\Sigma ,2}=d\phi_{A,2}+e\phi_{B,2}$$

Based on the linear variation of the conjugate phase with *l*, $\phi_{\Sigma ,2}$ can be rewritten as

(18) $$\begin{array}{cc} \phi_{\Sigma ,2} & =d\left(\frac{l_{2}}{l_{1}}\phi_{A,1}-2k_{A}\pi \right)+e\left(\frac{l_{2}}{l_{1}}\phi_{B,1}-2k_{B}\pi \right)\\ \; & =\frac{l_{2}}{l_{1}}\left(d\phi_{A,1}+e\phi_{B,1}\right)-\left(k_{\Sigma }\right)\pi \\ \; & =\frac{l_{2}}{l_{1}}\phi_{\Sigma ,1}-\left(k_{\Sigma }\right)\pi \end{array}$$

where $\phi_{A,1}$ and $\phi_{B,1}$ denote the conjugate phases of A and B, respectively, under interval $l_{1}$, $k_{A}$ and $k_{B}$ are the phase ambiguity integers, and $k_{\Sigma }=2dk_{A}+2ek_{B}$. By choosing moderate values of $l_{1}$ and $l_{2}$, $k_{\Sigma }$ is no longer a multiple of two. In this case, $\phi_{\Sigma ,2}$ can not be unwrapped correctly based on $\phi_{\Sigma ,1}$, while the single scatters still satisfies the linearity among different delayed intervals.

We therefore calculate the difference between the conjugate phases under $l_{1}$ and $l_{2}$ as

(19) $$\Delta \phi =\left|\phi_{2}-\left(\frac{l_{2}}{l_{1}}\phi_{1}-2k\pi \right)\right|$$

where $\phi_{1}$ and $\phi_{2}$ denote the conjugate phases of the scattering point under $l_{1}$ and $l_{2}$, respectively. When Δϕ is higher than the threshold $\eta_{\phi }$, the point will be considered to be a coincident point and be assigned to multiple extended targets.

#### 3.2.2. State Prediction and Update

The scattering points are detected from the range profile through the constant false alarm rate detection, which are input into the lower layer as measurements. The state vector of each measurement in frame *k* contains its distance r(k) and conjugate phase ω(k) and is denoted as

(20) $$X\left(k\right)={\left[r\left(k\right),\omega \left(k\right),\dot{r}\left(k\right),\dot{\omega }\left(k\right)\right]}^{T}$$

where $\dot{r}\left(k\right)$ and $\dot{\omega }\left(k\right)$ denote the first derivative of r(k) and ω(k), respectively.

The state transition model is written as

(21) $$\begin{array}{cc} \hat{X}\left(k+1|k\right) & =FX(k|k)+V(k)\\ \hat{P}\left(k+1|k\right) & =FP\left(k|k\right)F^{T}+Q\left(k\right) \end{array}$$

where $\hat{X}\left(k+1|k\right)$ and $\hat{P}\left(k+1|k\right)$ represent the predicting state and covariance matrix in frame k+1, respectively, X(k|k) and P(k|k) are the state matrix and covariance matrix after frame *k*, respectively, *F* is the transition matrix under a constant velocity model, and V(k) and Q(k) are the transition noise matrix and the process noise matrix, respectively.

Then, the tracker is updated as

(22) $$\begin{array}{c} K\left(k+1\right)=\hat{P}\left(k+1|k\right)H^{T}{\left[H\hat{P}\left(k+1|k\right)H^{T}+R\left(k+1\right)\right]}^{-1}\\ X\left(k+1|k+1\right)=\hat{X}\left(k+1|k\right)+K\left(k+1\right)\left[Z\left(k+1\right)-H\hat{X}\left(k+1|k\right)\right]\\ P\left(k+1|k+1\right)=\left[I-K\left(k+1\right)H\right]\hat{P}\left(k+1|k\right) \end{array}$$

where K(k+1) is the Kalman gain in frame k+1, *H* is the measurement vector, R(k+1) is the observation noise, Z(k+1) is the measurement in frame k+1, *I* is the identity matrix, X(k+1|k+1) and P(k+1|k+1) are the state matrix and the covariance matrix after updating, respectively.

#### 3.2.3. State Correction

Based on Equations (6) and (13), the averages of the radar distance and velocity of the scattering points in an extended target are calculated as

(23) $$\begin{array}{c} {\bar{R}}^{\prime }\left(t\right)=R+\cos\theta \left(t\right)\sum_{i=1}^{n}\frac{\Delta x_{i}}{n}\\ \bar{v}\left(t\right)=\frac{h(t)}{a}\frac{dh(t)}{dt}+d\sum_{i=1}^{n}\frac{\Delta x_{i}}{n} \end{array}$$

where *n* is the total number of scatters, $\Delta x_{i}$ is the distance between each scatter and the target center, and

(24) $$d=\frac{1}{a}\left[h\left(t\right)\cos\theta \left(t\right)\frac{d\theta (t)}{dt}-\sin\theta \frac{dh(t)}{dt}-R\sin\theta \left(t\right)\frac{d\theta (t)}{dt}\right]$$

When the distribution of scatters remains unchanged, $\sum_{i=1}^{n}\Delta x_{i}/n$ is constant. As a result, ${\bar{R}}^{\prime }\left(t\right)$ and $\bar{v}\left(t\right)$ are only related to the overall motion of the extended target. Therefore, the state of the tracking center $\left(r_{c},\omega_{c}\right)$ is regarded as the average state of scattering points and is calculated as

(25) $$r_{c}=\sum_{i=1}^{n}\frac{r_{i}}{n},\omega_{c}=\sum_{i=1}^{n}\frac{\omega_{i}}{n}$$

where $r_{i}$ and $\omega_{i}$ denote the radar distance and conjugate phase of the *i*-th scattering point, respectively.

The center of the extended target is tracked using the same KF framework as in Section 3.2.2. During the tracking process, the prediction values of the target center, ${\hat{r}}_{c}$ and ${\hat{\omega }}_{c}$, were used to correct the conjugate phases of the coincident points. The correction process was based on the linear variation of micro-motion with Δx, and each coincident point will be corrected to fit each extended target, respectively.

We assumed a coincident point to be distributed in the tracking gates of two extended targets at the same time and superimposed by two scattering points. For each extended target, if there exist more than two scattering points with correct conjugate phases, the states of the two nearest points, $\left(r_{1},\omega_{1}\right)$ and $\left(r_{2},\omega_{2}\right)$, will be carried out to calculate the revised conjugate phase as follows

(26) $$\omega_{r}=\omega_{1}+\left(r-r_{1}\right)\frac{\omega_{2}-\omega_{1}}{r_{2}-r_{1}}$$

where $\omega_{r}$ is the revised phase.

If only one scattering point has the correct phase, we calculate the difference as $\delta_{\omega }={\hat{\omega }}_{0}-\omega_{0}$, where ${\hat{\omega }}_{0}$ and $\omega_{0}$ denote the prediction and the measurement in the last frame, respectively. Then, the revised phase is calculated as $\omega_{r}=\hat{\omega }+\delta_{\omega }$, where $\hat{\omega }$ and ω denote the prediction and the measurement in the current frame, respectively. If no single scattering points exist, the predicted phases of the corresponding lower layer trackers will be used as the phases of the coincident points directly.

In this way, the conjugate phases of the coincident points were corrected and then used for state update process.

## 4. Results and Discussion

### 4.1. Simulation Results of Micro-Motion Extraction

**Figure 3:**
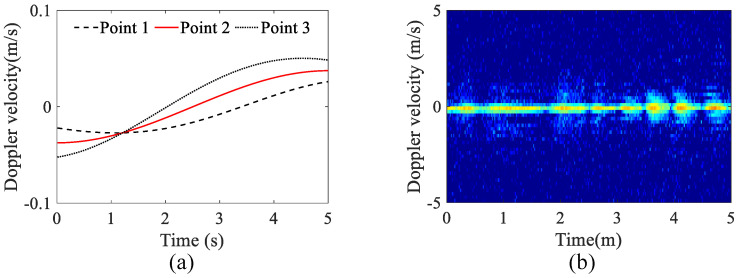
The theoretical micro-motion and the radar Doppler-time diagram of the vessel. (**a**) The theoretical velocity curves. (**b**) The simulated Doppler-time diagram of the vessel.

A vessel with no translation was considered in this simulation. The initial distance between the radar and the vessel center was 500 m. The vessel was composed of three scattering points, and Δx of the three points were −10 m, 0 m and 10 m, respectively. The motion parameters were set as follows: the heaving amplitude was 0.5 m, with a period of 10 s; the pitching amplitude was 1.5°, with a period of 10 s. The carrier frequency of the chirp pulse was 16 GHz, the pulse repetition time was 100 μs, the number of pulses in one frame was 512, and the bandwidth was 200 MHz. In this simulation, a sea clutter generation method considering the sea texture distribution, speckles, and sea spikes was adopted to generate the sea clutter echoes $n(t_{m})$ [21]. The theoretical velocity and the Doppler-time diagram of the vessel under a signal-to-clutter ratio (SCR) of 20 dB are shown in Figure 3a and Figure 3b, respectively. In Figure 3b, we noticed that the velocity variation caused by heaving and pitching was too small to be observed under the velocity resolution of 0.18 m/s.

**Figure 4:**
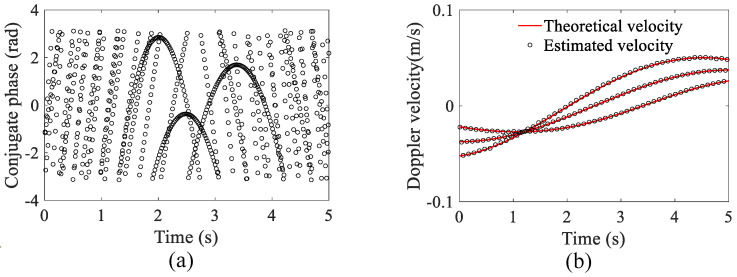
The result of MDCM method. (**a**) The initial phases of scattering points extracted from the radar range profile. (**b**) The estimated velocities derived from the conjugate phases by MDCM.

The initial phases of scattering points in each time step were extracted from the radar diagram and illustrated in Figure 4a. To unwrap the ambiguous phases, a 10-pulse MDCM was applied to the signal, and the conjugate phases were obtained, which were unwrapped and transformed into velocity values. The velocities derived from the conjugate phases of the simulated data with an SCR of 20 dB are plotted in Figure 4b. According to Figure 4b, the phase unwrapping was achieved effectively through the MDCM method, and the estimated velocity demonstrated good agreement with the theoretical velocity, with a root mean square error (RMSE) of 3.98 × 10${}^{-4}$m/s.

**Figure 5:**
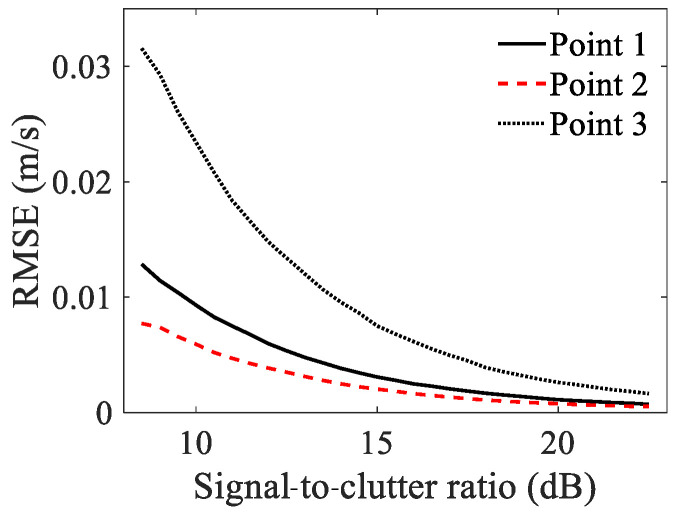
RMSE of velocity estimation with SCR of 8–23 dB.

Figure 5 presents the RMSEs of the velocity estimation with an SCR ranging from 8 dB to 23 dB. The phase measurement accuracy was directly influenced by the signal quality, which affected the velocity estimation results. Therefore, as the SCR increased, the RMSEs of all three points decreased. The RMSE of Point 3 was noticeably higher than that of Point 1 and Point 2, which was attributed to its lower scattering amplitude. When the SCR was above 15 dB, the RMSEs of all three points were less than 0.01 m/s, which is comparable to the wavelength and adequate for high-precision micro-motion extraction. We excluded the results with SCRs below 8 dB, because the RMSEs were extremely large, and the results could not reflect the real target velocity.

### 4.2. Simulation Results of Extended Target Tracking

**Figure 6:**
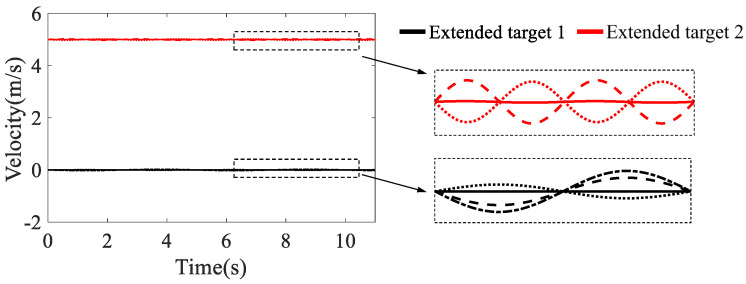
The theoretical velocities of the vessels.

We considered a radar detection scenario involving two extended vessels. The motion parameters of the two targets are listed in Table 1, while the theoretical velocities of the two targets are displayed in Figure 6. As is evident from the figure, the velocities of the scattering points of each target varied in a small range, which were difficult to discern from the Doppler-time profile with a decimeter-scale velocity resolution.

**Figure 7:**
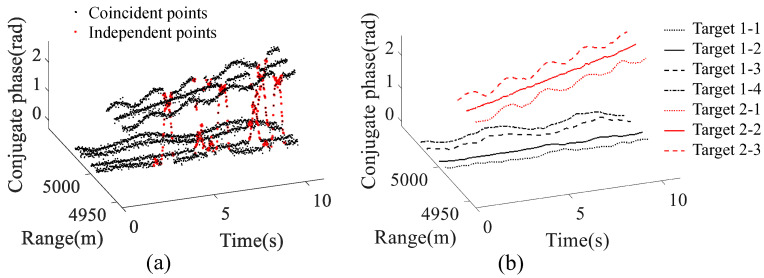
The simulated results of the proposed MDCM-LT algorithm. (**a**) The coincident points differentiated through the MDCM-based measurement partitioning method. (**b**) The tracking results derived through the LT algorithm.

The MDCM was applied to calculate the conjugate phases of the scattering points. We extracted the conjugate phases under 10-pulse delay ($\phi_{10}$) and 160-pulse delay ($\phi_{160}$), then calculated the difference between the linear converted phases ${\hat{\phi }}_{160}$ and $\phi_{160}$ as follows

(27) $$\begin{array}{cc} \Delta \phi & =\left|{\hat{\phi }}_{160}-\phi_{160}\right|\\ \; & =\left|\left(16\phi_{10}-2k\pi \right)-\phi_{160}\right| \end{array}$$

where *k* is the phase ambiguity integer. Δϕ was employed for coincident point classification, and the selected points are demonstrated in red in Figure 7a. As is seen in the figure, the linear variation of the conjugate phases among different delayed intervals *l* provides a novel way for identifying coincident points.

Subsequently, the tracking of the scattering points was accomplished by utilizing the proposed LT method. The tracking result of two targets moving near to each other is shown in Figure 7b. The black lines correspond to the four scattering points of extended target 1 (1-1, 1-2, 1-3, 1-4), while the red lines represent the three scattering points of extended target 2 (2-1, 2-2, 2-3). As can be seen from the figure, the shapes of the tracks conform to the theoretical velocity curves (Figure 6).

The tracking results of the proposed method were compared under various scenarios. The translational velocities of target 1 and target 2 were denoted by $v_{1}$ and $v_{2}$, respectively. In this simulation, $v_{1}$ was set to zero, while $v_{2}$ took values of −2, 0, 2, and 5 m/s. The RMSEs of the simulated scenes are shown in Table 2. The RMSE values indicate that the estimate accuracies of the ranges and velocities did not significantly change when the targets were moving near or away from each other, even when the scattering points of the two targets were coincident in the range dimension (when $v_{2}=$ 2 m/s or 5 m/s). These results demonstrate the effectiveness of the state correction process in the LT algorithm, which utilizes the motion correlation between scattering points and achieves the velocity estimation for the points without valid conjugate phase information.

**Figure 8:**
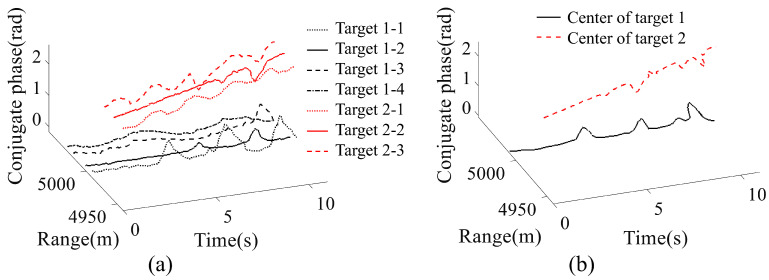
The simulated results of the comparison algorithms. (**a**) The scattering point tracking results of KF-RPRG method. (**b**) The center tracking results of CGT method.

The performance of the proposed MDCM-LT method was compared with the KF-ridge path regrouping (KF-RPRG) method [22] and the centroid group tracking (CGT) method [23]. The KF-RPRG method tracks individual scattering points and connects the interrupted tracks based on the gradient direction. The CGT method focuses on target centroids and classifies the target merging condition based on the number of measurements falling into the tracking gate of each target. The tracking results of the KF-RPRG and CGT are presented in Figure 8. In Figure 8a, *a*-*b* represents the *b*-th scattering points in the *a*-th extended target. It should be noted that the KF-RPRG does not differentiate which scattering point belongs to which extended target, and the black and red lines were only used for visual clarity in the plot. According to the figure, the KF-RPRG tracked the scattering points, but the fluctuations in the conjugate phases caused fluctuations in the movement trajectories. According to Figure 8b, the CGT offered stable tracking of the target centroids in both the range and velocity dimensions, but it was unable to track the micro-motion of each point.

The comparison between the three methods is listed in Table 3. The regrouping process of the KF-RPRG could only be performed once the tracking was completed, which limited the real-time trajectory output. Due to the CGT only tracking the centroids of the extended targets, its processing time was the shortest among the three methods. The proposed MDCM-LT method utilized the micro-motion disparities among scattering points to rectify the conjugate phases, which led to reduced fluctuations in the trajectory tracking results. Furthermore, the RMSEs of the extracted velocity and distance trajectories were held within 0.016 m/s and 0.277 m, respectively, thereby indicating the effectiveness of the proposed method.

### 4.3. Experimental Results of Extended Target Tracking

**Figure 9:**
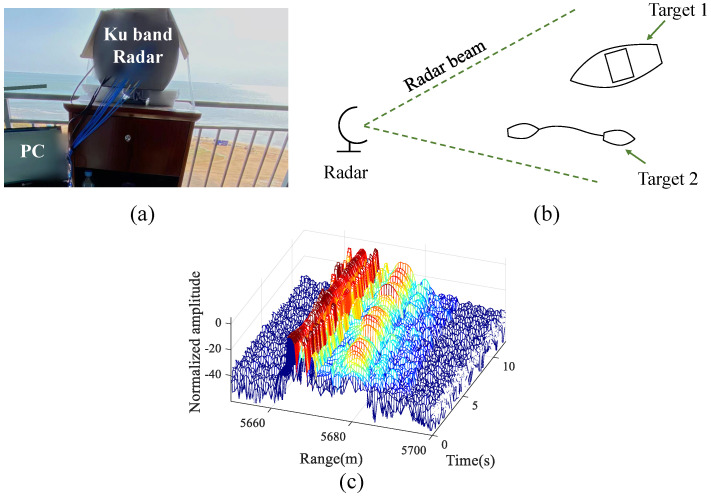
The experimental scenario. (**a**) The outdoor scene for the marine target detection experiment. (**b**) The positional relationship between the radar and targets. (**c**) The range–time profile of the experimental radar data.

A Ku-band radar was used for data acquisition in this experiment. The height of the radar placement was 60 m, and the distance between the radar and the targets was approximately 5600 m. In our experiment, extended target 1 was an anchored vessel. Extended target 2 consisted of two small ships connected by a rope, and the relative position between the two ships remained almost unchanged throughout the experiment. The experimental scene, the schematic diagram of target locations, and the range–time profile of the experimental radar data are illustrated in Figure 9.

**Figure 10:**
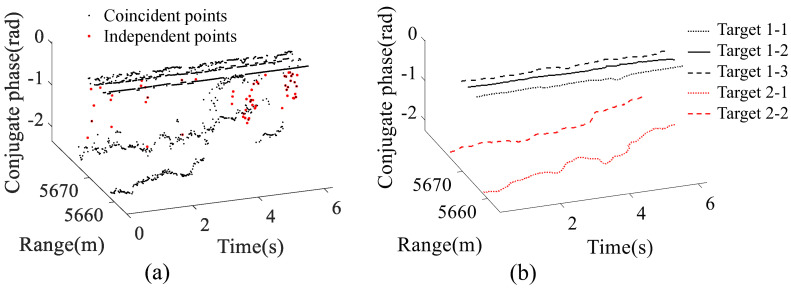
The experimental results of the proposed MDCM-LT algorithm. (**a**) The coincident points differentiated through the MDCM-based measurement partitioning method. (**b**) The tracking results derived through the LT algorithm.

Using delayed intervals of 6 and 10, the conjugate phases of the scattering points of two extended targets were extracted and used to classify the coincident points (as shown in Figure 10a). The LT method was then implemented for distance and conjugate phase tracking, with the tracking results presented in Figure 10b. In this figure, the black lines and red lines indicate the tracks of target 1 and target 2, while target 1 consists of three strong scattering points (1-1, 1-2, 1-3) and target 2 consists of two strong scattering points (2-1, 2-2). The conjugate phases were transformed into velocities, with the average velocities of target 1 and target 2 being −0.03 m/s and 1.56 m/s, respectively. The average velocity of the vessel scattering points was nearly zero, which agrees with its anchored state. The average velocity of small ships is in accordance with the values calculated from the translation in the range domain. In addition, the small ships showed shorter motion periods and larger motion amplitudes than the vessel, which is attributed to their lower mass. Consequently, the proposed method enabled us to successfully track the micro-motion of the extended marine targets when the targets overlapped in the range dimension of the radar echo.

## 5. Conclusions

In this paper, we proposed a MDCM-LT algorithm for extracting the trajectories of rigid marine targets from radar echoes. Using the proposed method, we extracted the high-precision micro-motion trajectories of overlapping targets on the wavelength scale. The LT took advantage of the motion association within rigid extended targets to improve the tracking performance. Additionally, the MDCM method offers a novel approach to classify overlapping extended targets, even when they have similar translational velocities. This approach could potentially be used for target classification in complex marine conditions, indicating potential avenues for future research in this field.

## Figures and Tables

**Table 1:** The parameters of simulated extended targets.

Parameter Name	Target 1	Target 2
Translational velocity (m/s)	0	5
Center position (m)	5000	4955
Δx of scattering points (m)	(−10, 0, 20, 30)	(−15, 0, 15)
Amplitude of heaving (m)	0.9	0.9
Period of heaving (s)	15	5
Amplitude of pitching (°)	3	3
Period of pitching (s)	10	5

**Table 2:** The RMSEs of range and velocity tracks.

$v_{1}$ (m/s)	$v_{2}$ (m/s)	RMSEs of Range Tracks (m)	RMSEs of Velocity Tracks (m)
0	−2	0.470	0.023
0	0	0.590	0.019
0	2	0.765	0.020
0	5	0.277	0.016

**Table 3:** The comparison between the KF-RPRG method, the CGT method, and the proposed MDCM-LT method.

Item	KF-RPRG	CGT	The Proposed Method
Group tracking	×	✓	✓
Scattering point tracking	✓	×	✓
Real-time output	×	✓	✓
RMSE of velocity (m/s)	0.062	0.028 (center)	0.016
RMSE of distance (m)	0.276	1.529 (center)	0.277
Processing time (s)	3.26	1.07	3.06

## Data Availability

The data presented in this study are available upon request from the corresponding author.
